# DVT as the Initial Presentation of Multiple Myeloma: A Rare Case Report and Literature Review

**DOI:** 10.7759/cureus.42600

**Published:** 2023-07-28

**Authors:** Sondos K Khalil, Leena Saeed, Abdalla Fadul, Mohamed F Elawad, Khaled Ferih

**Affiliations:** 1 Internal Medicine Department, Hamad Medical Corporation, Doha, QAT; 2 Radiology, Hamad General Hospital, Doha, QAT; 3 Medicine, College of Medicine Qatar University, Doha, QAT

**Keywords:** smoking, diabetes mellitus (dm), pulmonary embolism (pe), venous thromboembolism (vte), deep venous thrombosis, multiple myeloma

## Abstract

Multiple myeloma patients are recognized to have a higher risk of venous thrombosis. The cause of this could be attributed to several risk factors, such as circulating prothrombotic microparticles, disease-specific variables, and alterations in coagulation and fibrinolysis factors. Recent research has revealed that these individuals also experience greater arterial thrombosis, including acute myocardial infarction and stroke. In this case report, we present the clinical profile and management of a 42-year-old patient who presented with signs and symptoms of deep venous thrombosis (DVT) and was diagnosed with multiple myeloma. The aim of this case report is to highlight a rare clinical presentation and diagnostic workup in a patient with multiple myeloma. Additionally, we discuss the possible factors provoking the development of DVT as a first presentation before treatment initiation and their possible mechanisms.

## Introduction

Multiple myeloma (MM) is a malignant neoplasm involving the abnormal growth of plasma B lymphocytes. This disease commonly presents with bone destruction caused by the expansion of tumors, resulting in lytic lesions, bone pain, and sometimes fractures, which is often the initial diagnosis. Around 30% of patients initially show signs of hypercalcemia, which may be accompanied by renal failure due to the buildup of abnormal light chains in the collecting tubules. Approximately 10% of patients may experience symptoms such as hyperviscosity syndrome or bleeding disorders. Diagnosis usually involves identifying a spike in monoclonal protein levels through urine and blood tests, along with confirmation through bone marrow biopsy and immunophenotyping [1]. Thrombotic complications are commonly observed in both solid tumors and myeloid tumors. Tumor cells tend to promote blood clot formation. In the case of multiple myeloma, several specific mechanisms contribute to a state of increased blood clotting. Higher levels of immunoglobulins can directly inhibit the breakdown of blood clots. Inflammatory cytokines and other substances released during acute-phase reactions can trigger the process of blood coagulation. And finally, phospholipids may be targeted in an autoantibody fashion by the abnormal immunoglobulins [2].

Venous thromboembolism (VTE) complicating MM has been reported in the literature; however, it was mainly after treatment initiation [3,4]. Jeon et al. reported a case of a patient who presented with recurrent common cold and shortness of breath that eventually worsened; she was then found to have pulmonary embolism and deep vein thrombosis; nonetheless, DVT was not the initial or sole presentation [5]. In this report, we present a rare case of an undiagnosed MM presenting with leg swelling as the initial and sole symptom, diagnosed to have DVT accompanied by pulmonary embolism, which was found incidentally.

## Case presentation

A 42-year-old gentleman with a known case of diabetes presented to the emergency department after being referred from a private clinic with a case of right lower limb DVT. The history revealed right leg swelling and pain for one night; there was no history of trauma. He also complained of intermittent chest pain for the past four months and mid-thoracic back pain that was partially relieved with Ibuprofen but resumed a couple of hours later. No cough, hemoptysis, shortness of breath, or wheeze. The systemic review was significant for a sensation of irregular heartbeats, for which he sought medical advice and was advised not to start any medication. The patient is diabetic and on oral hypoglycemics, but he is not compliant with his medications. Past surgical history is significant for a pilonidal abscess drained surgically. The patient has a 30-year smoking history; he smokes one pack daily and consumes one can of alcohol daily on weekends. There is a family history of malignancy among his uncle and aunt. On examination, he was afebrile (37C), normotensive (136/59mmHg), RR 18 breaths/min, HR 99 beats/min, and maintaining saturation on room air. He was conscious, alert, and oriented to time, place, and person. Leg examination showed a swollen, tense, and warm right calf. CNS, cardiac, chest, and abdominal exams were all normal.

Initial investigations showed normal ECG and echocardiography. Comprehensive laboratory testing revealed an abnormal coagulation profile and blood chemistry. normal CBC, with peripheral blood smear showing normochromic normocytic red cells with increased rouleaux and shift to the left; no leukocytosis noted. Liver enzymes, renal function tests, and serum electrolytes were within normal ranges (Table 1).

**Table 1:** Laboratory testing showing abnormal coagulation profile and blood chemistry. INR: International normalized ratio, LDH: Lactate dehydrogenase, APTT: Activated partial thromboplastin time

Test	Value	Lab reference
Urea	6	(2.5-7.8 mmol/L)
Creatinine	100	(64-110 umol/L)
Sodium	136	(133-146 mmol/L)
Potassium	3.9	(3.5-5.3 mmol/L)
Adjusted calcium	2.44	(2.20-2.60 mmol/L)
Prothrombin time	13.1	(9.4-12.5 seconds)
INR	1.2	(critical high >4.5)
D-Dimer	14.36	(0.00-0.46 mg/L FEU)
APTT	28.8	(25.1-36.5 seconds)
Total protein	99	(60-80 gm/L)
Albumin	38	(35-50 gm/L)
LDH	393	(135-225 U/L)
HbA1C%	8.3	(≤ 6.0 %)

**Figure 1:**
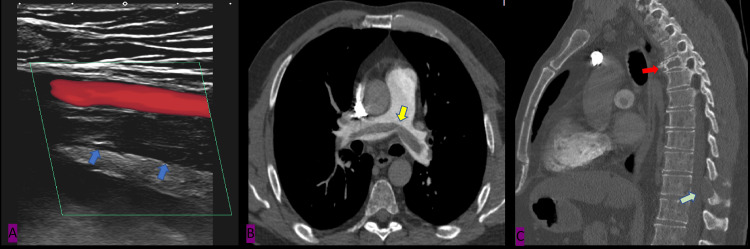
(A) Sagittal lower limb color doppler US shows thrombosed superficial femoral vein (blue arrow) extending to posterior tibial vein (Not shown), (B) Axial CT pulmonary angiogram demonstrates saddle pulmonary embolism (Yellow arrow), (C) Sagittal CT chest with bone reformat demonstrates T4 lytic lesion with compression fracture (Red arrow) and T10 posterior element lytic lesion (Green arrow) and multiple sternal lytic lesions.

Sagittal lower limb color Doppler US was done to confirm DVT diagnosis, and it exhibited a thrombosed superficial femoral vein extending to the posterior tibial vein (Figure 1A). CTA was done to rule out pulmonary embolism, which showed saddle pulmonary embolism (Figure 1B) and an incidental finding of lytic lesions in T4 and T10 suspicious of metastasis (Figure 1C).

He was sent to the ICU for treatment and monitoring. He was started on therapeutic enoxaparin and paracetamol. Laboratory testing and imaging were done to investigate the possibility of an underlying malignancy. Tumor markers, including CEA CA 19-9, and total PSA, were below the critical threshold for malignancy; however, beta-2-microglobulin was elevated; otherwise, autoimmune antibodies, factor V Leiden, and TB workup were unremarkable. MM-specific labs are shown below (Table 2).

**Table 2:** Multiple myeloma specific labs.

Test	Value	Lab reference
24-hour-urinary protein	0.23 gm/24hr	(0.03-0.15gm/24hr)
Urine protein electrophoresis and immunofixation	Presence of free kappa light chains	
Serum protein electrophoresis	IgG Kappa monoclonal band, 27.1 g/L	
KaFLC	299.1 mg/L	(3.3-19.4 mg/L)
LaFLC	8.7 mg/L	(5.7-26.3 mg/L)
Free ka/la index	34.38	(0.26-1.65)

A skeletal survey showed numerous osteolytic lesions in the skull, both scapulae, pelvic bones, and proximal right femur. He was then stepped down to the ward for the continuation of antithrombotic treatment, awaiting transfer to the oncology team for further workup and treatment.

**Figure 2:**
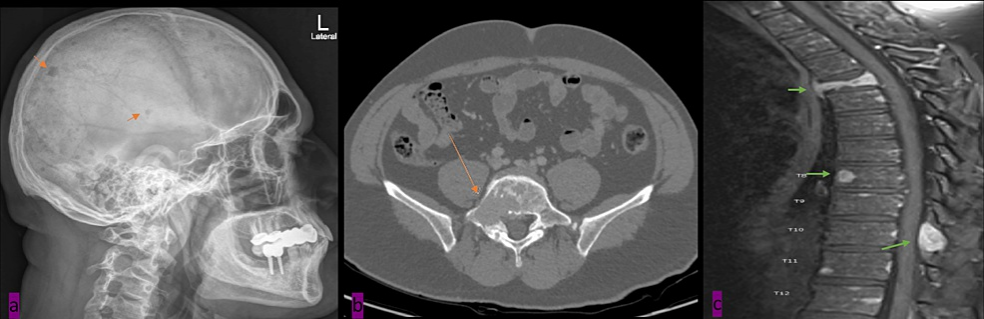
Multiple lytic punch-out bone lesions (Orange arrows) in the skull (a) and right sacral ala (b) multiple thoracic spine lytic lesions show enhancement in MRI T1-FS post-contrast (Green arrows).

**Figure 3:**
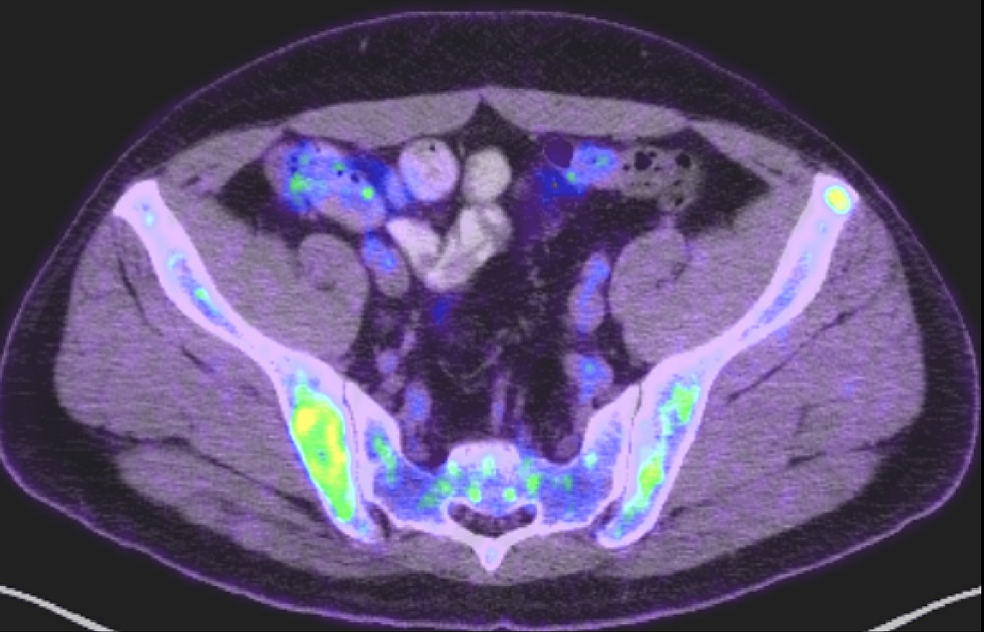
Whole-body FDG-PET/CT shows increased metabolic activity in multiple lytic skeletal lesions.

Upon transfer, further imaging and bone marrow aspiration were done. MRI images exhibited multiple lytic punch-out bone lesions in the skull (Figure 2a), right sacral ala (Figure 2b), and lytic lesions in MRI contrast of the thoracic spine (Figure 2c). In addition, whole-body FDG-PET/CT revealed increased metabolic activity in multiple body lytic lesions (Figure 3).

Bone marrow aspirate was cellular with adequate megakaryocytes, active erythropoiesis, reduced granulocytic cells, and infiltrated with myeloma cells comprising approximately 32%. Morphologically, it was mainly immature with some mature, binucleated forms and rare plasmablastic. Flow cytometry revealed 16% kappa monotypic plasma cells with aberrant expression of CD56. BM biopsy shows variable cellularity; most of the areas are hypocellular (~ 20%-35% cellularity), with a few other more cellular areas (~40%-55% cellularity) showing infiltration by kappa monotypic plasma cells; interstitial clusters, small sheets, and one intertrabecular space show extensive infiltration by the myeloma cells. By immunostaining, the plasma cells are aberrantly positive for CD56. The infiltration is roughly estimated at 40%-60% of the core cellularity. Reticulin stain shows no increase in reticulin fibers (MF0). Congo's red stain is negative.

An IgG kappa multiple myeloma, stage II diagnosis was reached. Upon the MDT meeting recommendation, he was started on the Dara-VRd protocol (daratumumab-bortezomib [Velcade], lenalidomide [Revlimid], and dexamethasone). He was discharged on rivaroxaban, insulin aspart, and insulin glargine for diabetes, valacyclovir, allopurinol, and co-trimoxazole. He completed six cycles of chemotherapy, which he tolerated well, and is scheduled for stem cell transplantation.

## Discussion

We are reporting a case of a Multiple Myeloma patient who presented with DVT before receiving immunotherapy. To the best of our knowledge, this is the first reported case of DVT as the initial presentation of MM in Qatar. Our literature review conducted in PubMed and the Cochrane Library revealed that no similar case had been reported. Patients with MM are at an increased risk of VTE, as is typical of any malignancy. The fundamental understanding of the mechanism of venous thrombosis is based on Virchow's triad, which comprises hypercoagulability, endothelial injury, and blood stasis. In cancer patients, all three components may be present.

The risk of VTE is heightened in individuals with MM who undergo treatment with immunomodulatory drugs (IMiDs), such as lenalidomide, or other thrombogenic drugs like dexamethasone [6-8]. A cohort study consisting of 2,397 individuals diagnosed with multiple myeloma between January 2007 and December 2013 found that the overall incidence of VTE at six months of treatment was 8.7% in patients who received lenalidomide treatment only, those administered concurrent dexamethasone, and those who received bortezomib [9]. Another study of 4,446 MM patients reported an incidence of 5.8% [10]. These findings provide important insights into the prevalence of VTE in MM patients receiving various treatments and highlight the potential for this complication in the early stages of treatment.

There are many hypotheses suggesting the mechanism of thrombosis in MM patients receiving IMiDs. One hypothesis suggested a transient decrease in thrombomodulin, which is an important cofactor in the anticoagulation pathway, during the first month of thalidomide therapy [11]. Another one proposed altering the expression of protease-activated receptor-1 (PAR-1) on endothelial cells, thereby increasing the risk of thrombosis, especially when thalidomide is combined with anthracyclines (e.g., doxorubicin) [12]. Furthermore, high levels of factor VIII and von Willebrand factor antigen were found in those treated with thalidomide, which are well-known factors associated with an increased risk of VTE [13]. Lack of anticoagulation factor activity has also been hypothesized thalidomide is associated with activated protein C (APC) resistance, which significantly increases the risk of DVT [14]. On the genetic level, genetic analysis revealed that the set of single nucleotide polymorphisms (SNPs) associated with thalidomide-related venous thromboembolism concentrated in genes and pathways that play a critical role in drug metabolism/transport, DNA repair, and cytokine balance [15]. Finally, the evidence indicates that IMIDs downregulate PU.1, resulting in a temporary blockage of maturation that leads to the accumulation of immature myeloid precursors and consequent neutropenia [16]. The aggregation of promyelocytes elevates platelet aggregation agonist and cathepsin G levels stored in azurophilic granules, which increases the risk of VTE [16]. The previously suggested hypotheses may also play a role in VTE incidence in MM patients before IMID initiation, which could explain the increased incidence of VTE following initiation compared to before treatment; however, further study is needed to find supporting evidence to validate this hypothesis.

Diabetes mellitus (DM) could be a potential cofactor in the risk estimates. The precise mechanism linking DM and VTE remains unclear, although a state of hypercoagulability in DM may be responsible [17]. Type 2 DM increases the risk for thrombus formation due to the suppression of fibrinolytic coagulation factor activity/level, creating a prothrombotic state [18]. On a cellular level, hyperglycemia and insulin resistance lead to increased PAI-1 production by responsible cells, a protein whose main function is blockage of t-PA action, thereby decreasing its activity and creating a thrombogenic state [19]. Our patient has uncontrolled diabetes with an HbA1C of 8.3, which could explain an enhanced coagulability state, increasing the risk for DVT in our patient.

Additionally, our patient has a smoking history of 30 pack years. Smoking is originally known as a major risk factor for VTE; however, a cohort study conducted in the municipality of Tromsø, Norway, by Enga et al. in 2008, which included 24,576 participants, revealed that there should be an additional predisposing factor for smoking to convey a risk of VTE; this includes cancer or smoking-related diseases [20].

Our case illustrates how hypercoagulability and VTE can be the initial presentations of MM. While the incidence of VTE is known to be higher in patients on IMID therapy, it’s important to consider other risk factors that may have an additive effect on the risk of VTE development.

## Conclusions

This case presents an atypical initial presentation of multiple myeloma. Although rare, practitioners should recognize its possibility of occurrence and report it. Various factors implicated in increasing the risk of VTE should be considered, reported, and studied to determine the incidence, prevalence, and disease burden of VTE before treatment initiation for a better understanding of the disease’s etiopathogenesis.
